# P-1649. Nursing Home Clinician Rationales Underlying Antibiotic Decision-Making for Suspected Urinary Tract Infection

**DOI:** 10.1093/ofid/ofae631.1815

**Published:** 2025-01-29

**Authors:** Sally Jolles, Christopher J Crnich, Lindsay Taylor, Robin Jump, Taissa A Bej, Corinne Kowal, Oteshia Hicks, Brigid Wilson, Madeline C Langenstroer, Deepthi Jacob

**Affiliations:** University of Wisconsin School of Medicine and Public Health, Madison, Wisconsin; University of Wisconsin School of Medicine and Public Health, Madison, Wisconsin; University of Wisconsin School of Medicine and Public Health, Madison, Wisconsin; VA Northeast Ohio Healthcare System, Cleveland, Ohio; Louis Stokes Cleveland VA Medical Center, Cleveland, Ohio; Louis Stokes Cleveland VA Medical Center, Cleveland, Ohio; VA Northeast Ohio Healthcare System, Cleveland, Ohio; VA Northeast Ohio Healthcare System, Cleveland, Ohio; University of Wisconsin School of Medicine and Public Health, Madison, Wisconsin; University of Wisconsin-Madison, Madison, Wisconsin

## Abstract

**Background:**

The factors underlying antibiotic decision-making in nursing homes (NHs) remains poorly understood. We conducted an analysis of discrete and free text responses from a mixed-methods survey presenting NH clinicians with two patient clinical vignettes on asymptomatic bacteriuria (ASB) and cystitis (CYS) designed to assess clinicians’ treatment (TX) threshold.

**Table 1:**
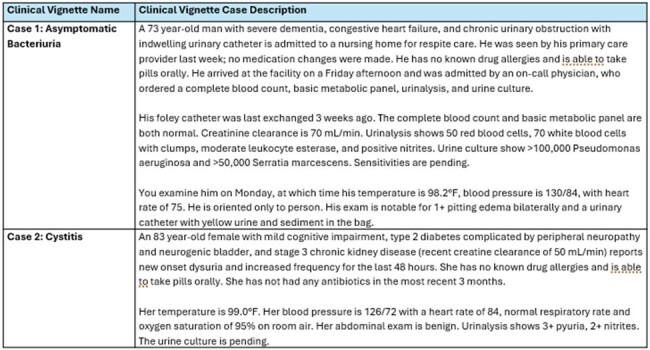
Clinical Vignettes

**Methods:**

NH clinicians were recruited nationally via professional organizations from December 2021 to April 2022. A web-based survey tool was used to administer the vignettes (Table 1) to participants who were asked to document their TX decision and justification. Treater (TRT) and non-treater (NTRT) responses were stratified by vignette and textual responses were uploaded to NVivo. A hybrid directed and conventional content approach was utilized to develop a codebook using the constant comparison technique.

**Table 2:**
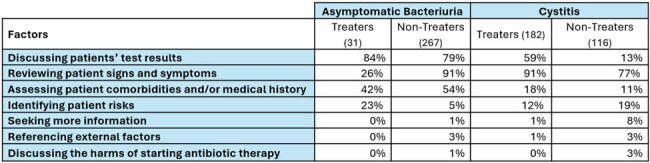
Factors Contributing to Clinicians’ Treatment Decisions by Vignette and Treatment Choice

**Results:**

Among 298 survey respondents; 10.4% treated ASB while 61.1% treated CYS. Seven factors contributing to clinicians’ TX decisions were identified (Table 2). For the ASB vignette (Table 3), TRTs framed the patient’s positive test results as infection (84%) while NTRTs framed it as contamination (79%). TRTs were less likely than NTRTs (26% vs 91%) to note the absence of signs and symptoms (S/S). Both groups cited the patient’s comorbidities and/or medical history (42% vs. 54%) in their TX decision. In the CYS vignette (Table 4), TRTs focused on positive patient S/S (91%) while NTRT did not feel there were sufficient frequency or severity of symptoms to justify immediate therapy (77%). TRTs were more likely to rely on positive test results (59%) and have more concern for patients’ comorbidities and/or medical history (18%).

**Table 3:**
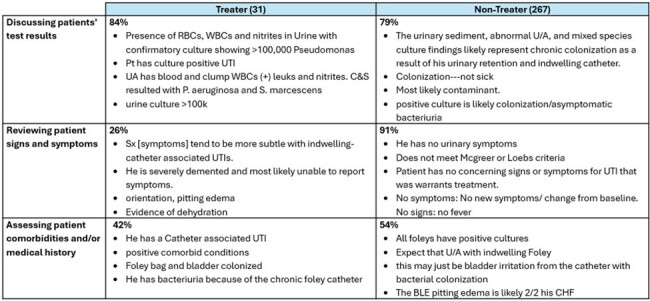
Asymptomatic Bacteriuria Vignette Illustrative Quotes

**Conclusion:**

Both TRTs and NTRTs use urine diagnostics in their decision-making, with TRTs having more confidence in the positive predictive value of urine diagnostics. NTRTs highlighted the lack of S/S as a justification for withholding therapy in both vignettes. TRTs accurately identified S/S for CYS, however, TRTs for ASB expressed concern that S/S might be underreported. This study shows how TRTs and NTRTs justify their rationales, however the next steps involve understanding how these decision-making patterns are amenable to education, information framing and/or risk management interventions.

**Table 4:**
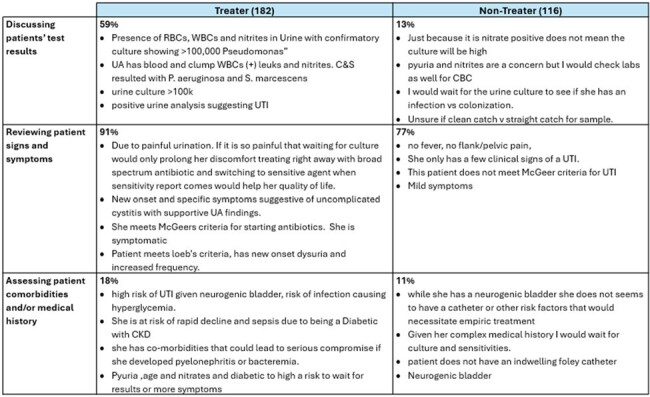
Cystitis Vignette Illustrative Quotes

**Disclosures:**

**Robin Jump, MD, PhD**, Abacus: Grant/Research Support|Merck: Grant/Research Support|Pfizer: Advisor/Consultant|Pfizer: Grant/Research Support

